# Competitive superhelical transitions involving cruciform extrusion

**DOI:** 10.1093/nar/gkt733

**Published:** 2013-08-22

**Authors:** Dina Zhabinskaya, Craig J. Benham

**Affiliations:** UC Davis Genome Center, University of California, One Shields Avenue, Davis, CA 95616, USA

## Abstract

A DNA molecule under negative superhelical stress becomes susceptible to transitions to alternate structures. The accessible alternate conformations depend on base sequence and compete for occupancy. We have developed a method to calculate equilibrium distributions among the states available to such systems, as well as their average thermodynamic properties. Here we extend this approach to include superhelical cruciform extrusion at both perfect and imperfect inverted repeat (IR) sequences. We find that short IRs do not extrude cruciforms, even in the absence of competition. But as the length of an IR increases, its extrusion can come to dominate both strand separation and B-Z transitions. Although many IRs are present in human genomic DNA, we find that extrusion-susceptible ones occur infrequently. Moreover, their avoidance of transcription start sites in eukaryotes suggests that cruciform formation is rarely involved in mechanisms of gene regulation. We examine a set of clinically important chromosomal translocation breakpoints that occur at long IRs, whose rearrangement has been proposed to be driven by cruciform extrusion. Our results show that the susceptibilities of these IRs to cruciform formation correspond closely with their observed translocation frequencies.

## INTRODUCTION

Although DNA *in vivo* is found mainly as a right-handed B-form helix, in principle it also can assume any of several other conformations. Some, such as the A-form and strand separated DNA, can occur in any base sequence, although the latter is favored in locally A + T-rich regions. Other structures either rigorously require or strongly prefer specific types of base sequence. These include the Z-form, which occurs at alternating purine–pyrimidine sequences; the cruciform, which requires a high degree of inverted repeat (IR) symmetry; the triple-stranded H-form, which needs long, mirror symmetric homopurine or homopyrimidine runs; and the four-stranded G-quadriplex structure, which needs four runs of G bases in proximity.

Conformational transitions from B-DNA to specific alternate structures can be induced by changes of temperature, ionic environment, hydration or superhelical state. The first three of these conditions are approximately constant *in vivo*; only the unrestrained DNA superhelicity is known to vary. Substantial levels of negative superhelicity are imposed on DNA by gyrase enzymes in prokaryotes and by transcriptional activity in all organisms (1). Although in eukaryotes this transcriptional superhelicity is transient, it is known to be substantial, generating a superhelix density of σ = −0.07, to travel over kilobase distances and to persist long enough to drive DNA structural transitions within this region (2). Recent work has shown that transcriptionally driven negative superhelicity travels between 1.5 and 2.5 kb from the site where it is generated (3,4). The latter investigation also found eukaryotic chromosomes to be partitioned into large-scale topological domains, with negative superhelicity occurring in domains where significant transcriptional activity was taking place.

Negative superhelicity imposes undertwisting torsional stresses on the DNA, which can drive transitions to alternate conformations that are less twisted in the right-handed sense than is B-DNA. As these transitions decrease the helical twist of the participating base pairs, they relieve some of the imposed superhelical stress. A transition will become favored at equilibrium when this free energy relief exceeds its cost.

*In vitro* experiments have demonstrated superhelical transitions from the B-form to each of several types of alternate structures, including Z-DNA (5,6), H-DNA (7), locally strand-separated DNA (8,9) and cruciforms (10–12). Although a structural transition was observed in a superhelical plasmid containing a quadriplex-susceptible region, it was not verified that this was the structure that formed (13). It has been suggested that this region might instead prefer to form H-DNA, to which it also is susceptible (14).

Several of these superhelically driven transitions also have been documented to occur *in vivo*. Z-DNA has been experimentally detected at inserted Z-susceptible regions in torsionally stressed bacterial DNA, both *in vitro* and *in vivo* (15–20). Indirect evidence suggests that Z-DNA also may occur in eukaryotic genomes *in vivo* (21–24). Superhelically driven regions of strand separation, a conformation that is required for the initiation of both transcription and replication, have also been detected *in vivo* (25,26). Recently, a technique called ssDNA-Seq has been developed that maps the unpaired regions that occur *in vivo* throughout a genome. This method found few open regions in transcriptionally quiescent cells. However, cells that are transcriptionally active were found to have many thousands of open regions. The average length of these regions was found to be ∼170 bp (27). They were located at sites which our SIDD theoretical analysis predicted would be most susceptible to superhelical denaturation. Transcriptionally driven cruciform extrusion has been shown to occur in introduced plasmids in *Escherichia coli* (28,29) and in yeast (30). Recently, regions of quadriplex DNA have also been found to occur in eukaryotic genomic DNA (31,32).

Given the highly polymorphic character of DNA, it commonly happens that multiple regions susceptible to transitions, possibly of different types, occur within the same superhelical domain. In this case, all possible conformations will compete for occupancy (33–36). This competition occurs because the relaxation caused by a transition anywhere in the domain will be experienced by, and affect the transition behaviors of, all other base pairs in that domain. For that reason, a rigorous analysis of superhelical transitions in genomic DNA must include multistate competitions.

Early theoretical methods to analyze superhelical transitions focused on simplified problems in which one or two susceptible sites were embedded in an otherwise resistant background (15,33,34,37,38). Later, more advanced statistical mechanical methods were developed that analyze the equilibrium of a superhelical genomic sequence in which all sites were susceptible to transition, but only one type of alternate structure was considered. Separate methods of this type were implemented to treat superhelical denaturation and B-Z transitions (39–41).

Recently, a unified model was developed to analyze competitions among multiple types of transitions in genomic sequences. Any transition could be included, provided its energetics was known. This approach initially was used to treat the competition between superhelical denaturation and B-Z transitions (42). Here we extend it to include superhelical cruciform extrusion at IR sequences, which may be either perfectly or imperfectly homologous. We have developed the DZCB*trans* algorithm (**D**enaturation, **Z**-DNA, **C**ruciform and **B**-DNA transitions) to analyze the superhelical equilibrium behavior of competitions involving these three transition types in kilobase-scale domains having any base sequence. We apply this method to analyze both cruciform extrusion in isolation and competitions involving denaturation, B-Z transitions and cruciform formation. We limit our consideration to these transitions because they are the only ones whose energies have been experimentally determined.

The initiation of cruciform extrusion from a twist-induced denatured bubble has been modeled theoretically using a dynamic coarse-grained Monte Carlo method (43). Although this treatment regarded the DNA as an isoenergetic homopolymer, it did capture important aspects of the dynamics of this nucleation event.

Cruciform extrusion behaves significantly differently from the other transition types considered here. Because both denaturation and B-Z transitions require additional free energy to extend a region of transition, their equilibria involve states in which considerable residual negative superhelicity remains. In contrast, once cruciform extrusion has initiated at a perfect IR, its extension is isoenergetic. This occurs because, although extension disrupts interstrand base pairs, it forms an equal number of identical intrastrand base pairs. The only energy change in this process comes from the incremental relaxation of superhelicity that occurs. For this reason, extension will be energetically favored to continue for as long as the sequence permits and negative superhelicity remains. The cruciform will extend either until the entire IR is involved or until the imposed superhelicity is fully relaxed.

Imperfections of symmetry in an IR can significantly alter its extrusion behavior. Symmetry violations must be accommodated within the cruciform arms by either mispaired or unpaired bases, both of which are energetically disfavored. So additional energy is required each time extrusion incorporates an imperfection. Depending on the energies involved and the level of residual superhelicity available, cruciform formation can be halted at an imperfection, even one as small as a single base insertion (44). When this happens, additional negative superhelicity is required to drive further extension.

IR sequences and their cruciforms have been proposed to serve various regulatory functions (45). Some efforts to investigate this possibility have relied on genome searches for IR sequences (46,47). That approach applied in eukaryotes found only short IRs to be enriched near transcription start sites (TSS). Because IRs may be present for several other purposes, such as RNA secondary structure formation or protein binding, these sequence-based methods cannot specifically identify those IRs that are involved in cruciform extrusion. Here we address this issue by analyzing the cruciform formation potential of IRs in transcriptional regulatory regions at the level of negative superhelicity to which they are subjected *in vivo*.

Cruciforms have also been suggested to be involved in pathological events, including chromosomal rearrangements (30). Specifically, a family of human translocations has been identified between palindromic AT-rich repeats (PATRRs) at locations 11q23 and 22q11 in the human genome (48–55). Experiments have detected high frequencies of these translocations in the germline DNA of healthy males. It has been hypothesized that cruciform formation at these IRs may be involved in the mechanism driving these translocation events. This possibility is supported by the observation that the majority of breakpoints are located in the spacer region separating the IR copies, which coincides with the loop region of the putative cruciform (56). Moreover, the sequences involved, when placed in superhelical plasmids, were found by atomic force microscopy to extrude cruciforms *in vitro* (53). Here we analyze the susceptibilities of these palindromes to adopt cruciforms in their genomic contexts at levels of superhelicity known to occur *in vivo*.

## MATERIALS AND METHODS

### Transition energetics

Consider a DNA molecule having a specified base sequence and imposed superhelix density σ. A conformational state of this molecule may contain regions in any of the three alternate conformations, Z-form, denatured or cruciform, with the remaining base pairs being in the B-form. The energy of this state includes the energies of transition to each alternate conformation, and the energy of superhelicity. The energetics associated with denaturation and B-Z transitions have been described elsewhere (39,41,42). The supercoiling free energy 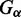 is a quadratic function of the residual superhelicity 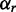, that part of the imposed linking difference α that remains after the fractional relaxation caused by all transitions (57):
(1)
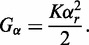



The value of the parameter *K* and the quadratic character of this superhelix free energy have both been determined experimentally (58,59).

The free energy *G_ex_* of cruciform extrusion consists of several components. First, there is an initiation energy, which comprises the cost of creating the Holliday junction between the cruciform and the interstrand duplex together with the cost of creating the two loops at the ends of the arms. The junction energy is regarded as constant, whereas the loop energy *G_l_* consists of the energy to melt each base pair in the loop, together with the loop entropy:
(2)
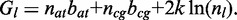

Here *n_at_* and *n_cg_* are the numbers of A/T and C/G base pairs in the loop, respectively, and *b_at_* and *b_cg_* are their corresponding melting energies. (Although this assumes copolymeric melting energies (60), near-neighbor energetics can be used instead.) The logarithmic term in this equation is the entropy associated with a loop of length *n_l_* (61). The factor 2 accounts for both loops in the cruciform, and the entropy parameter for DNA is 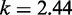 (62,63).

The initiation energy, as well as several other contributions to the cruciform energy to be described later in the text, has been determined from experiments that followed the extrusion of a long imperfect IR with a loop of 4 A/T bp (44). The extent of transition as a function of imposed superhelicity and temperature was determined by 2D gel electrophoresis. Because the temperature dependences of the free energies were assessed, they could be decomposed into their entropic and enthalpic components. The extrusion energy *G_ex_* of the perfect cruciform was found to have enthalpy of 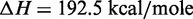 and entropy of 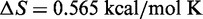. Because this extrusion energy was determined for a repeat with a loop of 4 A/T bp, the energy of extrusion for a perfect IR of any loop size is given by:
(3)
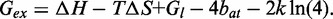



If an IR sequence is not perfect, its cruciform extends mispaired or unpaired bases must be incorporated into its arms. The energy cost of a mismatch has been found to depend on the identities of the mismatched bases and on their nearest neighbors (62). An insertion/deletion in the IR will cause a bulge in each of the cruciform arms. The energy required for bulge formation has been shown to depend only on the number of bases involved and their identities (44). Mismatches longer than a single base pair can be regarded as bulges.

### The competition among three transitions

As the energetics of cruciform extrusion are known, this transition may be incorporated into the multistate transition model that was developed previously (42). The resulting algorithm analyzes the equilibrium behavior of superhelical sequences in which cruciform extrusion, strand separation and B-Z transitions all compete.

The free energy *G* of a state in this competition consists of the energy costs of the three transitions, together with the total superhelical energy 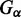. Suppose a state contains *r_d_* runs of denaturation, *r_z_* runs of Z-DNA and *r_c_* cruciforms. The total energy of this state is given by the following:
(4)
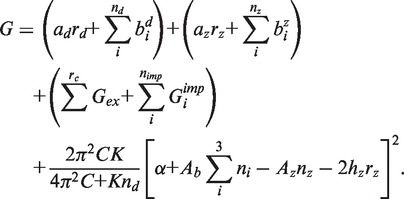

Here *a_d_* and *a_z_* are the nucleation energies for melting and B-Z transitions, respectively, and 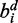 and 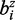 are their respective base-specific transition energies (39,41,42). The third term, the cruciform extrusion energy, consists of the extrusion cost *G_ex_* for each extruding IR, given by Equation (3), plus the energies *G^imp^* associated with all the imperfections they contain. The last term is the free energy associated with residual superhelicity when *n_d_* bases denature, *n_z_* transform into Z-form and *n_c_* form cruciforms. It follows that 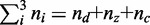 is the total number of transformed base pairs. The parameter *C* is the torsional stiffness coefficient, *A_b_* is the twist (turns/bp) of the B-form, *A_z_* is the twist of Z-DNA and *h_z_* is the twist at a B-Z junction (39,41).

### IR Finder (IRF)

We use the IRF algorithm developed by Gary Benson to identify sites with IR homology in DNA sequences (64). We assign parameter values designed to find all IRs whose properties make them potential candidates for extrusion. Because all base pairs in a loop must denature, loop energies increase with loop length. For this reason, we restrict the maximum loop size, which is the separation distance between the IR copies, to not exceed 100 bp. IR matches were scored 2, while mismatches and indels were assigned penalty scores of −10/bp. It was found that less stringent penalty scores for imperfections did not yield additional extrudable IRs. The threshold score for reporting an IR was set to be 20, so the shortest perfect IRs found had copy lengths of 10 bp. A simple calculation showed this to be the minimum length of a perfect IR that would have at least a 50% chance of extruding, if its loop region contained 4 A/T base pairs (the lowest energy loop), and it was the only transition possible in a 5 kb sequence at superhelix density σ = −0.06 and T = 310 K. These IRF parameters are conservative, only disallowing those repeats that have insignificant chances of forming cruciforms in an extreme case of a single perfect IR in a sequence background where no other transitions can occur.

As IRF treats sequences as linear, we analyze circular plasmids twice, with the start position of the second analysis moved to the middle of the original sequence. This strategy will find IRs that overlap the origin of the plasmid coordinate system.

When IRF finds an IR satisfying the aforementioned conditions, it reports the locations and lengths of its repeat copies, the loop length and the locations and types of all imperfections. If the loop found by IRF contains fewer than 4 bp, we incorporate its closest base pair(s) into the loop until its effective size becomes at least four. This is done because shorter loops are sterically difficult to form. This information is used to calculate the transition energetics of each cruciform, as described earlier in the text.

### The algorithm for analyzing three competing transitions

To analyze competitions involving superhelical melting, Z-formation and cruciform extrusion, we extend the previously developed multistate BDZ*trans* algorithm to include cruciform formation (42). This newly developed algorithm, which we call DZCB*trans*, uses the methods of statistical mechanics to calculate the equilibrium properties of a supercoiled DNA molecule whose sequence renders it susceptible to these three types of transitions.

Because exact computations are impractically slow to execute, an approximate approach is used. We consider a DNA domain having a specified base sequence, on which a set level of negative superhelicity is imposed. A state of this system is determined by specifying the conformation of each base pair and the twist experienced by any denatured regions. We first find the minimum energy state of this system and determine its free energy *E_min_*. Then we set an energy threshold above *E_min_*, and find all states whose energies are within that threshold (40).

We use the information from these states to construct an approximate partition function and to evaluate approximate values of all equilibrium properties of interest. These include the probability of each base pair experiencing each type of transition and the expected number of runs of transition of each type. The accuracy and execution time of this approach increase with the choice of threshold. However, extensive calculations have shown that thresholds can be selected for which this algorithmic strategy executes efficiently, yet produces highly accurate results (40). In practice, this algorithm commonly includes millions to billions of states of a 5 kb sequence, executes in seconds to several minutes and produces results that agree with exact calculations to four or more significant digits in all calculated parameters.

DZCB*trans* explicitly analyzes all states of extrusion, partial or complete, of every IR in the sequence, both perfect and imperfect. States of partial extrusion are included for two reasons. First, a long IR may entirely relax the imposed superhelicity while itself being only partially extruded. Second, under specific circumstances the extrusion of an imperfect IR can halt at a position where an imperfection is being incorporated into the cruciform arms (44).

In the calculations reported below, we use DZCB*trans* to analyze individual sequences 5 kb in length centered on sites of specific interest such as TSS. We adopt this length because superhelicity in eukaryotes has been shown to extend ∼1.5–2.5 kb from its point of generation (2–4). Therefore, choosing a region extending 2.5 kb in either direction from the site of interest accords with the biological scale of the phenomenon. Those calculations that assume a fixed value for the superhelix density commonly use 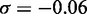, which is within the range found experimentally (2). This approach can be extended to treat sequences of any length by analyzing successive windows (40).

## RESULTS

### Properties of the three-way competition

First, we examine the superhelical competition between denaturation, Z-DNA and cruciform formation in a simplified yet illustrative situation. We analyze a 5000-bp sequence containing three separate regions, each highly susceptible to only one of the three types of transition. The denaturation-susceptible region is chosen to be (A)_60_, whereas the Z-susceptible region is (CG)_10_. The third region is a perfect IR with loop ACTG, left arm (C)*_n_* and right arm (G)*_n_*. To analyze the effect of IR length on this multistate superhelical competition, we compute at increasing values of the IR copy length *n*. The rest of the 5-kb sequence is chosen to be entirely G’s, hence is not susceptible to any of these transitions.

We use DZCB*trans* to calculate the equilibrium distribution among these transitions that occurs as the total IR length 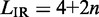 and the superhelix density σ are varied. We perform these calculations at temperature T = 310 K, for arm lengths 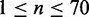 bp and superhelix densities 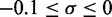.

**Figure 1.**
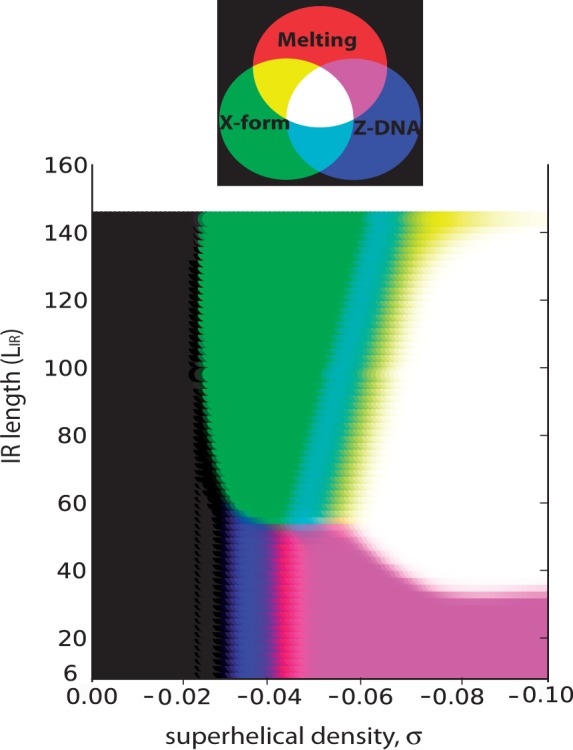
This color plot shows the probabilities of the three types of transitions as functions of IR length (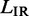) and superhelix density σ for the model sequence described in the text. The colors correspond to the probabilities of each transition type as shown in the Venn diagram at the top, with the depth of hue increasing with transition probability. Here X-form denotes the cruciform extrusion transition.

The results of this calculation are shown in Figure 1. The probability of transition to each of the alternate structures is depicted in a primary color—red for melting, blue for Z-DNA and green for cruciform formation. The intensity of the tone increases linearly with the transition probability. The color at each point on the plot describes the combined probabilities of the three types of transitions for that value of 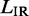 and σ. States in which more than one transition occurs at equilibrium are shown by mixed colors, as indicated by the Venn diagram at the top of the Figure. Black corresponds to no transition and white to all three transitions occurring simultaneously.

When 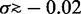, no transition of any type occurs at any length 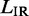. At more extreme negative superhelicities a complex competition occurs. For short IRs having 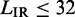, this competition involves only B-Z transitions and denaturation; cruciform extrusion does not occur regardless of superhelix density. The cruciform does not extrude under these conditions because the relatively small amount of superhelical energy relief it affords is not sufficient to compensate for its cost. The B-Z transition is the first to occur (blue), as the negative superhelicity σ becomes more extreme in this region of short IRs. At higher superhelix densities, a reversion from Z-DNA back to B-form is coupled to melting (red). As the superhelix density increases still further, both transitions occur simultaneously (purple).

For IRs with lengths in the interval 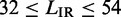, this same order of transitions is observed. In this situation, once the negative superhelix density has driven the other two transitions to completion, the cruciform also extrudes (white). A sudden qualitative change of behavior occurs for longer IRs, those with 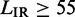. In this regime, cruciform extrusion dominates the other transitions. Here, it is the first transition to occur as the negative superhelicity increases (green). After extrusion is complete, the other two transitions occur in the same order as before: cruciform and Z-form (teal), then cruciform and melting (yellow) and finally all three transitions together (white).

This calculation documents several important properties of these competitions. First, under the assumed conditions, cruciform formation by moderate length IRs is not competitive with the other two types of transition. However, as IR length increases, cruciform formation becomes more probable until long perfect cruciforms dominate all other transitions. This example shows the complex interdependent manner in which these three transitions compete, which could not be captured by treating each transition separately. We note that the competitive behavior shown here can become more complicated for imperfect cruciforms, as extrusion may stop when an energetically costly imperfection is reached.

### Competing transitions near TSS

**Figure 2.**
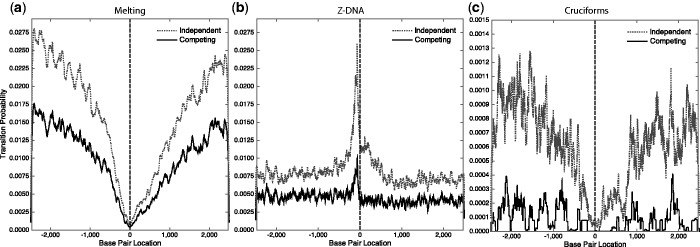
Average transition probabilities as functions of base pair location are shown for a set of 12 841 mouse genes that were aligned at their TSS (located at position 0) and oriented to transcribe to the right. These calculations were performed at T = 310 K and superhelix density σ = −0.06. Each panel shows results for a transition when it is analyzed in isolation, and when it competes with all others. Panel (**a**) shows denaturation, (**b**) shows the B–Z transition and (**c**) shows cruciform extrusion. The vertical scale is different for cruciforms compared with the other two transitions.

We examined the superhelical transition behavior of a set of 12 841 mouse gene sequences, each 5-kb long, centered at its annotated TSS and oriented to transcribe to the right. We used our numerical methods to calculate the transition probabilities for each of these sequences at T = 310 K and superhelix density σ = −0.06. Two methods were applied to analyze these sequences. First, each transition was analyzed in isolation, and, second, the competition among all three transition types was analyzed using the DZCB*trans* algorithm. In each case, the transition probabilities found at each location relative to the TSS are averaged over all the sequences in this data set. The results are shown in Figure 2.

In the competitive analysis, every sequence in this data set was found to undergo some sort of transition. Approximately 69% of them contain at least one region that denatures with probability *P* ≥ 0.5, whereas 59% contain site(s) that transform into Z-DNA with *P* ≥ 0.5. However, the frequency of cruciforms in this competition is extremely low. (The scale of the vertical axis is a factor of 20 smaller for cruciform formation than for the other two transition types.) Only 0.58% of the sequences contain IRs that extrude a cruciform with probability *P* ≥ 0.5 under these competitive conditions.

The same qualitative behavior is seen for each transition, whether analyzed alone or in competition. Both denatured regions and cruciforms tend to avoid the region around the TSS, whereas Z-forming sites cluster immediately 5′ of that location. However, treating each transition independently substantially overstates the number of transforming sites that are found in the competitive context. For denaturation, Figure 2a illustrates similar SIDD behavior for both methods close to the TSS, but at more remote locations the independent algorithm substantially overestimates the number of denaturing sites. Treating Z-DNA alone overestimates Z-forming sites everywhere in the 5-kb window, especially near the TSS. [A quantitative analysis of the superhelical competition between denaturation and Z-DNA has been presented elsewhere (42)] A clear pattern of depletion of extruding cruciforms around TSSs is observed in these mouse sequences when this transition is analyzed in isolation. But when competition is included, extruding cruciforms are barely present at all. These observations illustrate the complex non-linear character of superhelical transitions. In particular, the data in Figure 2 show that the three-way competitive behavior cannot be derived by combining the results from the three independent analyses.

We consider in greater detail the results involving cruciform extrusion that were derived from this mouse sequence set. When IRF was used with the parameters described earlier in the text to find all potentially cruciform-susceptible IRs in this set, it found 19 563 IRs in 9114 (70.9%) of these sequences. When cruciform extrusion was analyzed in isolation, only 1449 (7.4%) of these potentially susceptible IRs were predicted to extrude with probability *P* ≥ 0.5. These were located in 1431 sequences (11% of the total), 18 of which contained more than one such IR. But the analysis of the three-way competition found that only 75 sequences contain IRs whose probabilities of transition satisfy *P* ≥ 0.5, and only one sequence contains more than one such IR. That is, only 0.58% of the sequences in this data set contain competitively extruding cruciforms, and only 0.38% of the IRs found by IRF are actually competitive for transition under these conditions. This illustrates the potentially severe difficulties that can be encountered in attempting to infer transition susceptibility from sequence characteristics alone.

We consider further the set of 1449 cruciforms that were predicted to extrude when analyzed in isolation. As shown in Figure 2c, these extruding IRs occur with diminished frequency in the 1-kb region immediately surrounding the TSS. This depletion is not symmetric, extending somewhat further in the 3′ direction than in the 5′ direction. This shows that IRs capable of cruciform formation under physiologically reasonable conditions are infrequently present around TSSs in this mouse database, even when competing transitions are disregarded. Next, we partitioned this set into two subsets, depending on their extrusion probabilities in the competitive analysis. The 76 cruciforms that were competitive with melting and Z-DNA were found to be longer on average than the 1373 that were not competitive. The average total length of the competitive cruciforms was 71.4 bp, whereas those whose transition probabilities drop below 0.5 in the competitive analysis had an average length of only 48.1 bp. This result is consistent with those shown in Figure 1, where we found that cruciform extrusion became dominant at about 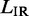 = 54 bp.

**Figure 3.**
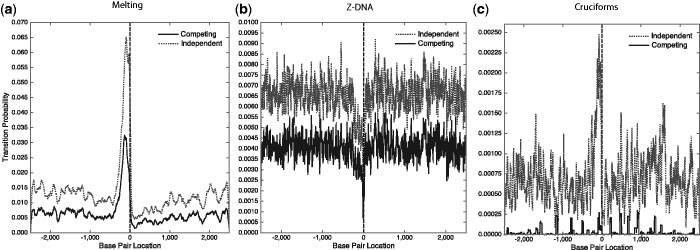
Average transition probabilities as functions of base pair location are shown for a set of 4442 *E. coli* gene sequences that were aligned at their start positions (located at position 0) and oriented to transcribe to the right. These calculations were performed at T = 310 K and superhelix density σ = −0.06. Each panel shows results for a transition when it is analyzed in isolation, and when it competes with all others. Panel (**a**) shows denaturation, (**b**) shows the B-Z transition and (**c**) shows cruciform extrusion. The vertical scales are different in each graph.

We also analyzed a database of 4442 *E. coli* gene sequences centered around their gene start sites. The same procedure and the same conditions were used in this analysis as in that of the mouse sequences. The results are presented in Figure 3. Interestingly, the patterns found in this prokaryote are opposite to those observed earlier for mouse. Denatured sites are strongly enriched in the 5′ flank of the *E. coli* genes, but highly depleted in mouse. There is a spike of Z-forming sites in the 5′ flank of the mouse sequences, but a similarly sharp depletion in *E. coli*. When analyzed alone, sites extruding cruciforms are depleted around the TSS in mouse but sharply enriched in the immediate 5′ flanks of *E. coli* genes. Our analysis, excluding competition, found that 20.6% of these genes have one or more IRs with extrusion probabilities *P* ≥ 0.5, twice the frequency found in the mouse sequences. This suggests a difference in the cruciform formation proclivities of eukaryotes and prokaryotes. However, in both organisms the susceptibility to cruciform extrusion is vastly diminished by competition with the other transition types. When competition with denaturation and Z-DNA is allowed, only 0.25% of the *E. coli* genes contain a cruciform with extrusion probability *P* ≥ 0.5. In this genome, most cruciforms are outcompeted by strongly denaturing sites near the TSS (42).

These results suggest that superhelical cruciform formation may be involved in the transcriptional regulation of a small number of mouse genes, but could be more widely used in *E. coli*. In mouse, not only are there few sequences containing sites that are capable of undergoing this transition in competition with others but those that do occur avoid the region immediately surrounding the TSS. Therefore, the IRs that are present in these sequences may serve other purposes than the extrusion of cruciforms.

### Chromosomal translocations

As long IRs dominate the transition behavior of superhelical sequences, their extrusion could be biologically important in either normal or pathological processes. Long IRs may be vulnerable to mutations and have been implicated in specific translocation events. A PATRR region on human chromosome 11 (PATRR11) has been shown to occur in several mutated versions, which participate in translocation events with different frequencies. Moreover, the region on chromosome 22 to which translocation occurs is also a PATRR (PATRR22). This translocation event is denoted t(11;22). *De novo* t(11;22) translocations involving these regions have been detected by PCR in sperm DNA from normal males (50,51,54,55). Both breakpoints in these translocation events occur in the region separating the repeat copies, which form single-stranded loops in their extruded cruciforms. This suggests that cruciform formation may be involved in the mechanism of this translocation. Substantial indirect evidence supports this possibility (53). The different alleles of the IRs involved, when placed in superhelical plasmids, have been shown by atomic force microscopy to extrude cruciforms. Moreover, the *de novo* frequency with which each PATRR11 allele participates in a translocation event correlates with its *in vitro* ease of cruciform formation. To study this question further, we analyzed the competitive extrusion potential of each allele of both PATRR11 and PATRR22 in their genomic context, over a range of superhelix densities known to occur *in vivo*.

The polymorphic variants of PATRR11 have been classified into three groups according to their *de novo* translocation frequencies and sequence characteristics. The L-PATRR11 group contains two symmetric long IRs that have high translocation frequencies. The SS-PATRR11 group contains four symmetric relatively short IRs with intermediate translocation frequencies, whereas the AS-PATRR11group contains eight asymmetric shorter IRs with low translocation frequencies (54,55). The L-PATRR11 IRs range from 445 to 450 bp in length, whereas those in the SS-PATRR11 and AS-PATRR11 groups vary from 63 to 434 bp in length. In general, the symmetric IRs tend to have shorter loop regions with arms that are nearly perfectly matched, whereas the asymmetric IRs have longer loops and more imperfections. Average values for these parameters over the three groups are shown in Table 1.

**Table 1.** Properties and average competitive cruciform formation probabilities 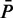 for the three classes of PATRR11 alleles

Type	L-PATRR11	SS-PATRR11	AS-PATRR11
Number of IRs	2	4	8
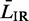 (bp)	447.5	359.5	278.6
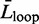 (bp)	4	10.75	41
Match (%)	98.4	98.9	95.7
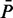 (σ = −0.03)	0.83	0.55	0.006
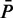 (σ = −0.04)	1.0	0.99	0.17
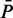 (σ = −0.06)	1.0	1.0	0.39

If the mechanism of these t(11;22) translocations involves cruciform extrusion, as has been proposed, one would expect that the *in situ* susceptibilities to cruciform formation of the alleles in these groups would correspond to their translocation frequencies. To investigate this question, we use DZCB*trans* to evaluate the competitive cruciform formation abilities of these alleles under standard conditions in their genomic contexts.

We located the most common PATRR11 sequence in human chromosome 11, then selected for analysis the 5000-bp segment that is centered on that IR. Next, we replaced this standard allele with each of the other known alleles, keeping the lengths of all analyzed sequences fixed at 5 kb. We used DZCB*trans* to analyze the competitive transition behaviors of these sequences at T = 310 K and three levels of supercoiling spanning the range of expected *in vivo* values. We calculated the probability of each type of transition, including the cruciform extrusion probability of each allele. The results of these calculations, averaged over the alleles in each of the three classes, are presented in Table 1.

The three groups show substantially different average competitive cruciform formation probabilities 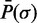 as functions of superhelix density. The L-PATRR11 alleles are the easiest to transform, occurring largely or exclusively in the extruded state at every level of superhelicity examined. The SS-PATRR11 alleles are less susceptible to extrusion at low superhelix densities, but comparable with the L-PATRR11 group when 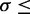 −0.04. The different susceptibilities found at σ = −0.03 occur because the SS-PATRR11 alleles have longer loops, hence higher initiation energies. So the onset of their transitions is delayed to more negative superhelix densities. IRs in these two groups clearly dominate denaturation and Z-formation in the competition for superhelical transition.

The last allelic group, AS-PATRR11, was found to be largely resistant to cruciform extrusion. Even at σ = −0.06 their average probability of extrusion is only 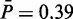. This means that these IRs are unlikely to form cruciforms at the levels of superhelicity achieved *in vivo*. Our calculations indicate that this behavior occurs because the IRs that comprise these alleles are not competitive with the other types of transitions. In particular, because they are AT-rich, we find that under superhelical stress they prefer to denature rather than to form a cruciform.

One of the AS-PATRR11 sequences (Accession No. AB235190) behaves in an unexpected manner. Its repeat contains 434 bp, with a 5-bp loop and a 94.55% IR homology, properties that should conduce to cruciform formation. As it is long, nearly perfect and has a low energy cost for loop formation, one would expect it to belong to the L-PATRR11 group that has high translocation frequencies and cruciforms that dominate all competition. However, it was placed in the AS-PATRR11 group because its *de novo* translocation frequency was found to be low.

Our calculations find that the cruciform extrusion probability of the genomic sequence containing this allele is low. Even at the extreme superhelicity of 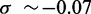, its cruciform formation probability is near 0. The reason for this apparently anomalous behavior lies in the details of its sequence. Although this IR contains only 12 imperfections in a 215-bp-long arm, nine of these imperfections are clustered within 40 bp of the loop, while the IR symmetry beyond this point is nearly perfect. So to extrude a cruciform beyond this point, a large energy barrier must be overcome. Even if initiated, extrusion would halt at these imperfections. Substantial increments of negative superhelicity would not be sufficient to drive extension beyond this region. As this partially extruded cruciform would be relatively short, it is not competitive with the other transitions. As this IR is also 93.76% AT-rich, it has a strong competing tendency to denature. Our calculations show that denaturation outcompetes cruciform formation in this sequence at all superhelix densities up to 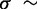 −0.08, beyond the level that has been observed in eukaryotes.

We also examined the three PATRR22 alleles that are the t(11;22) translocation partners found on chromosome 22. All three alleles were observed to have similar translocation frequencies, comparable with those seen in the L-PATRR11 group (55). Our calculations also show that they have comparable extrusion behaviors. The IR in each allele extrudes with high probability, even at low superhelix densities. (Results not shown.)

These results show that the translocation frequencies of these alleles do correlate with their *in situ* cruciform formation properties. This in turn supports the contention that cruciform extrusion may be involved in the mechanisms of these translocation events.

### Translocation-susceptible sites in human Chr1

We next assessed the frequency in the human genome of IRs with the attributes of length, homology and loop size that were shown earlier in the text to correspond with translocation potential. To this end we used IRF to search human chromosome 1 for IRs, using the same parameters as were described in the ‘Materials and Methods’ section. IRF found 104 466 IRs in this 249 Mb chromosome, an average of one every 2.4 kb. We then assessed the transition behavior of each of these IRs in isolation. This was done by inserting each into a background that was all G’s, so the total sequence length was 5 kb. We then used DZCB*trans* to evaluate their transition behaviors at T = 310 K and σ = −0.06.

We found that 1556 of these IRs have extrusion probabilities satisfying *P* ≥ 0.5. That is, even without competition from the other sequences in their genomic contexts, only 1.5% of the IRs found by IRF will extrude cruciforms. As the cruciforms implicated in translocation events have long arms and short loops, we assessed these attributes in this subset of 1556 extruding IRs. Only 81 of these IRs have total lengths 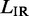 exceeding 100 bp, and loop sizes <20 bp, an average of one every 3 Mb. This low frequency may be a consequence of the instability of these regions, as shown by the mutability of the PATRR11 sequences, and by the deleterious character of the translocation events they could potentiate. In light of these two attributes, it is reasonable to expect that long IRs with significant translocation potential would be sparse in the human genome.

## DISCUSSION

In this article, we have developed and applied computational methods to analyze cruciform extrusion in negatively superhelical DNA molecules having any base sequence, both in isolation and in competition with strand separation and B-Z transitions. These methods allow for imperfections in the IR symmetry and include partially extruded states.

As shown here, cruciform formation behaves qualitatively differently from the other transitions with which it competes. This is because, once extrusion of a perfect IR has initiated, its extension does not require any additional transition energy. So extrusion will proceed to completion, which can be either extruding the full IR or relaxing all the imposed superhelicity. In contrast, because both B-Z transitions and denaturation require additional energy to extend a region of transition, they reach equilibrium in states that still have substantial residual superhelicity. However, the initiation cost of cruciform formation is higher than that of either B-Z transitions or denaturation. So the onset of transition depends on the properties of the cruciform. A short perfect IR will be unable to compete with the other two transitions at any physiologically attained superhelix density, as it will not relieve enough superhelicity to overcome its higher initiation cost. But the competitiveness of extrusion increases with IR length, other factors remaining fixed, until cruciform formation at long IRs dominates the transition behavior. The superhelix density at which cruciform extrusion becomes competitive depends on its initiation energy, which varies with the length and base composition of its loop region.

The presence of imperfections in the IR symmetry complicates this picture because incremental energy is needed to incorporate the resulting bulges and/or mispairings into the cruciform arms. Extrusion can halt at appropriately placed imperfections, even ones as small as a single base indel (44). However, these obstructions to extension can be overcome when the negative superhelix density becomes more extreme. Once this happens, extrusion will proceed either to completion or to the next imperfection. The transition behavior of an imperfect IR also depends strongly on the number and placement of its imperfections. An imperfection is more likely to obstruct extension the further it is from the loop region. This happens because the amount of superhelicity that has been relaxed before the imperfection is encountered becomes larger, so there is less superhelical free energy remaining to overcome the obstruction. However, multiple imperfections close to the loop region can prevent extrusion from being competitive because they collectively constitute a high energy barrier that must be overcome before the extension can relax substantial superhelicity. This behavior was seen in the anomalous AS-PATRR11 sequence analyzed earlier in the text.

We analyzed 5-kb regions around the TSS of 12 841 mouse genes to assess the prevalence of IRs susceptible to cruciform formation in those regions. An analysis in which this transition was the only one permitted found that 1431 of these sequences contain IRs that extrude cruciforms under physiological levels of negative superhelicity. And when competition with strand separation and B-Z transitions was included, only 75 of these sequences were predicted to extrude cruciforms with probability *P* ≥ 0.5. Qualitatively similar results were found in the analysis of 4442 *E. coli* genes. However, the patterns of transformation near gene start sites showed opposite tendencies for each transition type in this prokaryote and in the mouse sequences.

Although IRs occur frequently in genomic DNA, few of them appear to be able to produce competitive cruciforms under physiological conditions. Our analysis shows this to occur in at most ∼0.6% of the mouse gene sequences examined. Moreover, the few cruciform-capable IRs that were found in this analysis appear to avoid the region around the TSS. This suggests that cruciform extrusion may be involved in the transcriptional regulation of at most a small proportion of genes. Interestingly, IRs that are too short to extrude cruciforms are enriched immediately upstream of TSSs in eukaryotic genomes (46). Some of these sites have been verified to be transcription factor binding motifs. This reinforces the observation that many IRs may be present in genomic DNA for reasons other than cruciform extrusion.

Most efforts to find genomic sites that can occur in alternate conformations simply search for locations with the required sequence attributes. Our results show that this approach applied to cruciform formation at IRs yields unacceptably high number of false positives. Although IRF finds 19 563 susceptible IRs in the analyzed mouse gene set based on their sequence characteristics, only 1449 of these are predicted to extrude in isolation. So this search produces thirteen times as many false positives as putative true positives. Moreover, only 76 of these IRs extrude in competition with strand separation and B-Z transitions, which is 0.38% of those found in the sequence search. This shows that simple sequence searches, and to a lesser degree equilibrium analyses that do not consider competitions with other transition types, can greatly overestimate the number of sites that have the potential to transform under imposed superhelicity.

The measured translocation frequencies of different alleles of the IR PATRR11 and PATRR22 sequences that participate in a t(11;22) translocation event were shown here to correspond with their *in situ* competitive cruciform formation abilities. This strongly supports the proposal that the mechanism of this translocation may involve cruciform extrusion. The behavior of the anomalous allele in the AS-PATRR11 group is particularly supportive of this view. This sequence is long, highly A + T -rich, with few imperfections and a short loop, all attributes that should favor cruciform formation. However, the imperfections in this sequence are clustered near the loop region, an arrangement which our analysis shows makes extrusion non-competitive with denaturation. This example further illustrates the importance of analyzing superhelical transitions in competition rather than individually.

Analysis of human Chr1 shows that long IRs with the attributes that correlate with translocation ability in the PATRR sequences occur infrequently in the human genome. This may be due in part to negative selection pressures on such sequences caused by their susceptibilities to mutation and translocation.

The algorithmic strategy presented here for analyzing multistate competitive transitions can easily be modified to incorporate additional types of transitions as their energetics become known. At present, the only transitions for which this information is available are strand separation, B-Z transitions and cruciform extrusion. Our model shows that the competition among these types of transition presents a rich repertoire of complex behaviors. As G-quadriplexes and H-DNA triplex structures also have been suggested to be biologically important (31,32), we look forward to incorporating them into our analysis once these energetics have been experimentally characterized.

To date, only a few experimental investigations have been reported into mechanisms by which superhelical transitions and protein binding events conspire together to regulate specific genes (25,65). The systematic investigation of the occurrences and putative functions of alternate DNA structures *in vivo* presents important experimental challenges and opportunities. A full understanding of these phenomena requires a high-resolution view of the distribution of superhelicity throughout chromosomes, and how this changes with cell type, during development, through the cell cycle, with protein and nucleosome binding or release and with transcriptional activity, among other factors. It also requires the development of techniques to find each type of alternate structure without perturbing the system in ways that affect its propensity to form.

Recently, some promising experimental techniques have been reported that address these requirements. A method to assess the *in vivo* level of superhelicity throughout a genome has found eukaryotic chromosomes to be partitioned into large-scale topological domains, with negative superhelicity occurring in domains where significant transcriptional activity is taking place (4). Also, the ssDNA-Seq technique has identified a large number of unpaired regions that occur *in vivo* in the genomic DNA of transcriptionally active cells (27). Moreover, sites which our SIDD computations predict would be most susceptible to strand separation were found by ssDNA-Seq to be open *in vivo*. These open regions were long, the most frequent length being ∼170 bp. The authors claim their ssDNA-Seq method can also find other alternate structures that have open base pairs. These specifically include Z-form regions, because their B-Z junctions are disordered, and the loop regions of cruciforms. So experimental information on the global occurrence of competitive transitions such as are analyzed here should be available soon.

Much of the current information regarding *in vivo* alternate DNA structures is informative, but indirect. For example, evidence of a structure is found by inserting the putatively susceptible sequence into a plasmid, inserting this into a cell and finding *in vivo* evidence consistent with its transition. Although such results are important indicators of transition susceptibility, they do not prove conclusively that the given transition occurs at this sequence when in its natural genomic context. Certainly the competition this site experiences would be different in the two contexts, and the levels of superhelicity imposed on it may also differ. Until methods are available that simultaneously find all transitions occurring in a DNA sequence, the theoretical technique presented here provides the only means to understand how a proposed superhelical transition that has been experimentally investigated in isolation would act in its genomic context when competing with the other transitions to which that region is susceptible.

In this article, we analyzed the extrusion of the cruciforms that are proposed to facilitate the translocation that causes Emanuel syndrome, a severe neurological disease. This is just one instance where DNA secondary structure has been posited to play a role in human disease. Although the exact mechanisms that create genomic instabilities are not well understood, non-B DNA structures have been implicated in various disease processes, many involving chromosomal rearrangements. The breakpoint sites for numerous chromosomal translocations are known to have sequences that are susceptible to forming alternate DNA secondary structures (66,67). Cruciforms in particular have been also implicated in polycystic kidney disease (68). Understanding the thermodynamics of competing superhelical transitions may yield new insights into a variety of pathological events. Our model provides the only way presently available to predict the behavior of such sites in their genomic contexts. As such, it may prove useful in guiding experimental investigations of these important processes.

Sequences may be submitted to our Web site http://benham.genomecenter.ucdavis.edu for automated analysis by the DZCB*trans* algorithm. The sequence must be either in FASTA format or in a file that contains sequence characters exclusively. Sequences of any length up to 10 kb may be submitted, although sequences of length ∼5 kb are preferred. The user can request analysis of any combination of the three transition types, denaturation (SIDD), B-Z transitions (SIBZ) and/or cruciform extrusion, at any level of superhelicity.

In the near future, we hope to analyze the competitive transition characteristics of a large number of genomic sequences, up to and including complete genomes. The results will be posted in a database on our Web site.

## FUNDING

National Science Foundation [DBI-0850214]. Funding for open access charge: Grant DBI-0850214 from the National Science Foundation.

*Conflict of interest statement*. None declared.
